# Autophagy Protects Monocytes from *Wolbachia* Heat Shock Protein 60–Induced Apoptosis and Senescence

**DOI:** 10.1371/journal.pntd.0003675

**Published:** 2015-04-07

**Authors:** Vijayan Kamalakannan, Abijit Shiny, Subash Babu, Rangarajan Badri Narayanan

**Affiliations:** 1 Centre for Biotechnology, Anna University, Chennai, Tamil Nadu, India; 2 Madras Diabetes Research Foundation and Dr. Mohan's Diabetes Specialties Centre, Chennai, Tamil Nadu, India; 3 National Institutes of Health—National Institute for Research in Tuberculosis-International Center for Excellence in Research, National Institute for Research in Tuberculosis, Chetpet, Chennai, Tamil Nadu, India; University of Liverpool, UNITED KINGDOM

## Abstract

Monocyte dysfunction by filarial antigens has been a major mechanism underlying immune evasion following hyporesponsiveness during patent lymphatic filariasis. Recent studies have initiated a paradigm shift to comprehend the immunological interactions of *Wolbachia* and its antigens in inflammation, apoptosis, lymphocyte anergy, etc. Here we showed that recombinant *Wolbachia* heat shock protein 60 (rWmhsp60) interacts with TLR-4 and induces apoptosis in monocytes of endemic normal but not in chronic patients. Higher levels of reactive oxygen species (ROS) induced after TLR-4 stimulation resulted in loss of mitochondrial membrane potential and caspase cascade activation, which are the plausible reason for apoptosis. Furthermore, release in ROS owing to TLR-4 signaling resulted in the activation of NF-κB p65 nuclear translocation which leads to inflammation and apoptosis via TNF receptor pathway following the increase in IL-6 and TNF-α level. Here for the first time, we report that in addition to apoptosis, rWmhsp60 antigen in filarial pathogenesis also induces molecular senescence in monocytes. Targeting TLR-4, therefore, presents a promising candidate for treating rWmhsp60-induced apoptosis and senescence. Strikingly, induction of autophagy by rapamycin detains TLR-4 in late endosomes and subverts TLR-4-rWmhsp60 interaction, thus protecting TLR-4–mediated apoptosis and senescence. Furthermore, rapamycin-induced monocytes were unresponsive to rWmhsp60, and activated lymphocytes following PHA stimulation. This study demonstrates that autophagy mediates the degradation of TLR-4 signaling and protects monocytes from rWmhsp60 induced apoptosis and senescence.

## Author Summary

Despite knowing the significance of *Wolbachia* in helminth infections, our understanding of immunity and pathogenesis remains incomplete. Therefore, considering the gravity of the problem, the present study provides evidence that *Wolbachia* heat shock protein 60 induces apoptosis and senescence through TLR-4. Also, binding of rWmhsp60 to TLR-4 triggered caspase cascade activation following, ROS-mediated mitochondrial potential loss. Moreover, we found that nuclear translocation of NF-κB p65 was predominantly related to TLR-4 expression and resulted in apoptosis- and senescence-mediated inflammation via TNF-α and IL-6. Hence, we hypothesized that modifying TLR-4 expression may provide a plausible target for designing antiparasitic drugs. Here we have shown that induction of autophagy by rapamycin destabilizes TLR-4 expression and protects monocytes from rWmhsp60-induced apoptosis and senescence. In addition, rapamycin-induced monocytes were unresponsive to rWmhsp60 and triggered lymphocyte activation after PHA stimulation. Thus, synergistic usage of rapamycin with existing anti-filarial drugs might reduce the TLR-mediated inflammatory reactions following microfilaricidal treatment.

## Introduction

Lymphatic filariasis is a debilitating parasitic infection with nematodes *Wuchereria bancrofti* and *Brugia malayi*, associated with varied clinical outcomes, such as lymphedema, hydrocele, or elephantiasis [1]. Chronicity by such metazoan parasites often foists crippling morbidity and incapacitating disability with profound economic, social and political consequences [2]. Abundant data from the literatures suggest that immunopathological reactions that play a major role in the development of filarial infection result from the cumulative effects of inflammation evoked mainly by the microfilarial stage of worms that invade tissues [3].

The most enthralling aspect in disease pathogenesis is individuals with subclinical conditions have compromised antigen-specific T-cell responsiveness with diminished proliferation and IFN-γ expression in response to antigen stimuli [4] and altered functions of antigen-presenting cells (APCs) [5–7]. Antigen-mediated T regulatory mechanisms are supposed to be potentiated majorly by the APCs. Impaired production of regulatory cytokines (IL-12 and IL-10) by APCs [6], monocyte dysfunction [8] and apoptosis [9] has been reported earlier during filarial infection. Furthermore, monocyte function of the patients is diminished based on adherence, spreading and phagocytic efficiencies in response to a bacterial stimulus [10]. Hence, failure of APCs function has been implicated to underlie this T-cell unresponsiveness.

Filarial parasites rely on endogenous *Wolbachia* for embryogenesis, growth, survival and also contribute to pathogenesis of filarial disease [11]. Outbreak of disease pathogenesis after host inflammatory response provoked by death of the parasite and severe systemic inflammation following chemotherapy are attributed to the release of *Wolbachia* into the circulation [12]. Few reports suggest that *Brugia malayi* and *Onchocerca volvulus* extracts induce inflammation while the parasites that cause rodent filariasis devoid of *Wolbachia* failed to induce inflammation [13]. Also extracts from Wolbachia found to replicate these inflammatory effects [13]. These reports imply the crucial role of *Wolbachia* exposure in immune responses origination and the development of filarial pathology. Similarly, adverse reactions during microfilaricidal treatment has been associated with increase in inflammatory IL-6 and TNF-α [12] production by APCs where toll like receptor-4 (TLR-4) signaling appeared to be operative [14]. Hence, the possibility that the *Wolbachia* antigens such as LPS, surface protein, heat shock protein 60 (hsp60), CpG motifs and peptidoglycan may play a role in immune regulation that necessitates investigation. Apart from its ascribed primary function as intracellular molecular chaperone, heat shock proteins also elicit a potent pro-inflammatory response and, therefore, has been proposed as a danger signal of stressed or damaged cells [15–17]. Both human and bacterial hsps are found to stimulate and regulate innate and acquired immune responses during pathogenesis that leads to severe autoimmune disorders [18] and chronic inflammation [19,20]. In this perspective, *Wolbachia* hsp60 has been shown to evoke IgG1 antibody response in chronic patients [21]. In addition, we earlier have shown that *Wolbachia* hsp60 induces pro-inflammatory cytokine production and apoptosis in monocytes [9] and T-cell unresponsiveness, [22] a hallmark status during filarial infection. Until now, endotoxin-like molecules, present in the nematode extracts, were thought to be the major inducers of these responses [23]. A recent study has found the striking similarity of innate immune responses to human hsp60 and LPS [24,25]. Few reports even suggest that like LPS, hsp60 can interact with TLR-2 and TLR-4 [26] and provoke inflammation and apoptosis. Similar observations were also documented in filarial conditions, where filarial and Wolbachial extract exhibit interaction with TLR-2 and TLR-4 [23]. Despite considerable evidence that Wmhsp60 can evoke pronounced innate immune response, the mechanisms and the pathways responsible remains unknown.

In the present communication, we demonstrate the molecular mechanism that underlies rWmhsp60 induced monocyte dysfunction and T-cell unresponsiveness, more importantly, the involvement of TLR-4 signaling. In addition, the present study has highlighted rapamycin, a clinically approved drug-induced autophagy as one of the inherent methodology to subvert inflammation provoked by monocyte apoptosis and senescence by limiting rWmhsp60–TLR-4 interaction. This strategy might even strengthen the current Mass Drug Administration (MDA) program by using rapamycin synergistically with diethylcarbamazine citrate (DEC).

## Methods

### Study population

Ten asymptomatic amicrofilaremic endemic normal (EN) and five individuals with active infection and/or chronic lymphatic pathology (CP) were included in this study. Standardized histories were obtained and physical examinations were done on all the participants in and around Chennai, India, an area endemic for *W*. *bancrofti* infection. Patients were recruited through Apollo Hospitals, Chennai, India, after obtaining informed consent with protocols approved by the Institutional Review Board of the Anna University. All the individuals were screened for the presence of circulating filarial antigens by Og4C3 mAb ELISA, a marker of *W*. *bancrofti* infection and adult worm burden [27] (TropBio, Townsville, Australia) (Table 1).

**Table 1:** Characteristics of the study population: Demographic details of the individuals included in the study.

Group	Male/Female	Median age	Range (years)	Clinical manifestations	Treatment	Mf load (mf/ml)	CFA Og4C3 (IU)
Endemic normals (10)	6/4	30	25–35	None	None	None	None
Chronic patients (5)	3/2	45	40–55	Grade II–IV lymph edema	DEC/heat therapy	None	None

Note: Mf—Microfilaria; CFA—Circulating filarial antigen.

### Isolation of peripheral blood mononuclear cells and monocytes

Peripheral blood mononuclear cells (PBMCs) were isolated from the study population by density gradient centrifugation with Pancoll from Pan-Biotech (Aidenbach, Germany). Cells were washed in RPMI 1640 medium, containing HEPES 25 mM and 80 mg gentamicin for 10 min at 1200 rpm and resuspended in the medium supplemented with 10% fetal calf serum (Pan-Biotech). Monocytes were then purified from the upper interface of a hypotonic Percoll density gradient (1.129g/mL). Purified monocytes were resuspended in RPMI 1640 medium and the purity was found to be at least 90% as assessed by fluorescent microscopy using FITC-conjugated antihuman CD14 antibody. Finally, PBMCs or purified monocytes were stimulated with 100 ng/ml of LPS (Sigma) or 10 mM of cyclohexamide or 5μg of rWmhsp60 for 24 h and cultured in a humidified 5% CO_2_ incubator at 37°C.

### Production of recombinant Wmhsp60

Recombinant *Wolbachia* heat shock protein 60 (rWmhsp60) was expressed and purified as described previously [21]. In brief, *Wolbachia hsp60* gene was PCR amplified from *B*. *malayi* genomic DNA and cloned in pRSET-A vector for expression of the recombinant protein. Recombinant plasmid was then transformed into BL21 (DE3) *Escherichia coli* host, and the expression was induced using 1 mM IPTG, followed by purification using immobilized metal affinity chromatography. Assessment of endotoxin contamination was done using a Limulus amoebocyte lysate assay, which showed <1 pg of LPS/10 mg of protein.

### Cytotoxic effects of rWmhsp60

Dose–response study was carried out with normal healthy volunteers to determine the optimum cytotoxic dose of rwmhsp60 using 3-[4, 5-dimethylthiazol-2-yl]-2, 5-diphenyl tetrazolium bromide (MTT) from Life Technologies (Waltham, Massachusetts, USA). PBMCs and monocytes were stimulated with various concentrations of rWmhsp60 (1, 2, 5, 10 and 20 μg/ml) for 48 h. After treatment, cells were incubated with MTT (10 μl, 5 mg/mL) at 37°C for 4 h and then with DMSO at room temperature for 1 h. The plates were read at 490 nm on a scanning multiwell spectrophotometer. As the optimum concentration was found to be 5 μg/ml of rWmhsp60, MTT assay was again performed in the presence and absence of polymyxin B sulphate, to check the endotoxin contamination.

### Assessment of rWmhsp60-induced apoptosis in PBMCs of EN and CP

PBMCs (10^6^/ml) from EN and CP were seeded in 24-well culture plates, stimulated with rWmhsp60 and 100 mM Cyclohexamide (CHX) from Sigma (St. Louis, USA) and incubated for 24 h. CHX-treated cells served as positive control. After 24 h, the cells were then pelleted at 4°C (500×g) for 5 min, washed twice with PBS, and resuspended in 100 ml of 1× annexin-V–binding buffer (0.1 M Hepes/NaOH at pH 7.4, 1.4 M NaCl, 25 mM CaCl_2_) with 2 mg/ml of annexin-V-FITC (BD Biosciences, San Jose, CA, USA) and 2 mg/ml of propidium iodide (PI) (Merck, NJ, USA). Finally, cells were washed and resuspended in 2× PBS, acquired on a Becton Dickinson FACS Calibur (BD Biosciences) and analyzed using Cell Quest software (BD Biosciences). For acquisition, forward and side scatter gates were adjusted to acquire monocytes and lymphocytes separately, and the data analysis was performed using FlowJo software (Tree star, San Carlo, CA).

### Caspase-3 activity assay

Caspase-3 activity was measured in isolated monocytes of EN following rWmhsp60 and CHX treatment. The activity of caspase-3 was calculated from cleavage of the fluorogenic substrate AC-DEVD-AMC from EMD Millipore (Billerica, Massachusetts, USA). After 24 h incubation, rWmhsp60- and CHX-treated cell lysates were incubated with substrate solution (caspase-3 substrate AC-DEVD-AMC 20 mg/mL, HEPES 20 mM, glycerol 10%, dithiothreitol 2 mM, pH 7.5) for 1 h at 37°C, and cleavage of the caspase-3 substrate was measured at an excitation wavelength of 390 nm and an emission wavelength of 460 nm. Activity was expressed as percentage fold increase in relative fluorescence unit (RFU).

### Isolation of cell extracts and Western blotting

For analysis of caspase cascade and autophagic events, total cell lysates were prepared as reported previously [28]. Briefly, cells were treated with rWmhsp60, CHX for apoptosis experiments, and with rWmhsp60 in the presence and absence of rapamycin for autophagy experiments. After 24 h incubation, cells were lysed with 50 mM Tris–HCl (pH 8.0), 150 mM NaCl, 1% Triton X-100, and 100 μg/mL phenylmethylsulfonyl fluoride and 1 μg/mL aprotinin. Proteins were separated by SDS/PAGE (15% polyacrylamide), transferred to a nitrocellulose membrane, blocked with 5% milk powder in TBST (50 mM Tris, 150 mM NaCl and 0.05% Tween 20, pH 7.4) and probed with the appropriate antibody. Blots were developed by the NBT/BCIP substrate (nitrobluetetrazolium salt/5-bromo 4-chloro 3-indolyl phosphate; USB Cleveland, OH, USA). For NF-κB experiments, monocytes were pre-incubated in the presence and absence of N-acetyl cysteine (NAC) (Sigma) for 30 min before the addition of rWmhsp60 or LPS. Cells were harvested after 24 h, and the remaining steps are carried out as described earlier.

### Determination of ROS and mROS superoxide levels

To visualize total intracellular levels of ROS and mROS superoxide, immunofluorescence assay was performed in monocytes of EN. Log-phase cells were grown on 24-well plates and treated with rWmhsp60 or CHX as indicated. The culture medium was removed, and the cells were washed with PBS and incubated with CM-H_2_DCFDA from Life Technologies (to measure the total cellular H_2_O_2_ levels) Life Technologies at a final concentration of 2.5 mM and/or MitoSOX (to measure the mitochondrial superoxide levels) (Life Technologies) at a final concentration of 5 mM in serum-free RPMI1640 for 30 min at 37°C. For immunofluorescence assay, the cells were mounted with ProLong Gold Antifade Reagent with DAPI (Life Technologies), and images were acquired using a LSM-710 confocal microscope (Carl Zeiss, Jena, Germany). For fluorescent spectrometer analysis, cells were treated with DCF2-DA, digested, and analyzed using 495 nm excitation and 527 nm emission filters.

### Determination of mitochondrial membrane potential

Mitochondrial membrane potential changes were determined by the uptake of tetramethylrhodamine, ethyl ester (TMRE) (Life Technologies) fluorescence. After treatment, monocytes were harvested and incubated with 1 μM TMRE at 37°C for 15 min in the dark. Cells were washed and resuspended in PBS and analyzed using 550-nm emission filter in UV fluorescent spectrometer.

### RNA extraction

After 24 h treatment with rWmhsp60 and CHX, monocytes were lysed, and the total RNA was extracted according to the RNeasy Mini kit manufacturer’s protocol (Qiagen, New Delhi, India). RNA was dissolved in 20 ml of RNase-free water.

### cDNA synthesis

Reverse transcription of RNA was performed in a final volume of 40 ml containing 0.25 mM mix of the 4 deoxynucleotide triphosphates (dATP, dGTP, dTTP and dCTP) (New England Biolabs, MA, USA); 1 reverse transcriptase buffer (50 mM Tris–HCl, pH 8.3, 75 mM KCl, 3 mM MgCl_2_); 8 mM DTT; 20 U RNase inhibitor (Life Technologies); and 200 U of MMLV–reverse transcriptase (New England Biolabs) followed by incubation of the tubes at 37°C for 60 min. The reverse transcription reaction was stopped by heating the tubes at 90°C for 5 min. The cDNAs were snap chilled in ice for 5–10 min and stored at 20°C until use.

### Real-time RT-PCR

Real-time quantitative RT-PCR was performed in an ABI 7500 sequence detection system (Life Technologies) using TaqMan Assays on Demand reagents for Bcl-2, Bax, Bid, Bad, GAPDH, and an endogenous 18S ribosomal RNA control. The end point used in real-time PCR quantification is CT that is the threshold cycle during the exponential phase of amplification, according to the manufacturer’s protocol. Quantification of gene expression was performed using the comparative CT method (Sequence Detector User Bulletin 2; Life Technologies) and reported as the fold change relative to the housekeeping gene. To calculate the fold change, the CT of the housekeeping gene (18S rRNA) was subtracted from the CT of the target gene to yield the ΔCT. Change in the expression of the target gene as a result of antigenic exposure was expressed as 2^-ΔΔCT^, where ΔΔCT = ΔCT of stimulated − ΔCT of unstimulated. Along with fold change, basal level expression of the same genes was also assessed.

### Immunofluorescence assay

Monocytes from EN were plated on the cover slips and cultured at up to 50–60% confluence. After treatment, cells were washed with PBS, and fixed with fresh 4% paraformaldehyde solution for 15 min at room temperature. Cells were then washed twice with PBS, followed by incubation in 10% normal rabbit serum–blocking solution for 20 min at room temperature in a humidified chamber. Cells were incubated with specific primary antibodies against NF-κB p65 (Cell Signaling Technologies, Danvers, MA, USA) for 2 h at room temperature in a humidified chamber. Cells were washed 3 times in PBS and incubated with Alexa Fluor 488–conjugated goat anti-rabbit IgG (Life Technologies) for 45 min at room temperature in a humidified chamber. The cells were then washed in PBS, mounted with ProLong Gold Antifade Reagent with DAPI (Life Technologies). Images were acquired using Carl Zeiss LSM-710 confocal microscope with 10 fields of view. Above-mentioned protocol is also followed for TLR-4 surface expression studies with TLR-4 (Life Technologies) and LC-3 (Cell signaling Technologies) specific antibodies.

### ELISA

Monocyte culture supernatants for cytokine profiling was recovered after 24 h stimulation and kept frozen until batch analysis. The levels of cytokines IL-6 and TNF-α in the culture supernatants were measured conventional by ELISA following manufacturer’s protocol (Life Technologies). The results were expressed as fold change over control. The concentration of interferon-γ was determined using an IFN-γ Quantikine enzyme-linked immunosorbent assay kit (R&D Systems, Minneapolis, MN, USA) according to the manufacturer’s instructions.

### Protein–protein docking

The crystal structures of human TLR-2-TLR-1 (2Z7X) complex, TLR-4 (3FXI) and TLR-9 (4QDH) were acquired from the Protein Data Bank. *Wolbachia* hsp60 structure was obtained from The Protein Model Portal database designed with *E*. *coli* GroEL (1FYA) as template. TLR and Wmhsp60 structures were submitted as receptor and ligand, respectively, to HEX and ClusPro, protein–protein docking servers for complex prediction. The analysis was conducted using a default parameter. Interprosurf was further used for refinement and global energy values scoring, and the models with lowest energy were selected for further analysis. UCF Chimera software was used to plot predicted intermolecular interactions.

### TLR surface expression

After 24 h treatment of monocytes with rWmhsp60, cells were incubated with saturating amounts of anti-TLR2-FITC or anti-TLR4-APC or anti-TLR9-PE and isotype-matched nonbinding control mAb as per manufacturer’s protocol. All the antibodies were purchased from eBiosciences (San Deigo, CA, USA). Cells were then acquired on a FACSCalibur (BD Biosciences) and analyzed using Kaluza software.

### Blocking studies

For blocking experiments, monocytes were cultured for 2 h, in the presence of anti-TLR-2 or TLR-4 or TLR-9 antibody or rapamycin (50 nM). After this time, cells were treated with rWmhsp60 for additional 24 h, and monocyte apoptosis was analyzed in FACS Calibur using annexin-V/PI staining technique, described previously.

### Observation of autophagy by monodansylcadverine staining

Monocytes (5×10^5^ /well) were cultured in 24-well culture plates. After 24 h of incubation with rWmhsp60 or rapamycin or both, the cells were washed with 2× PBS and incubated with 0.05 mmol/L monodansylcadverine (MDC) (Sigma) at 37°C for 1 h, and the change in fluorescence was observed by Eclipse TS100 fluorescence microscope from Nikon (Tokyo, Japan) at an excitation wave length 380 nm with an emission filter of 525 nm.

### Senescence-associated β-galactosidase staining

Sa-β-gal activity was detected as previously described [29]. Cells were washed once with PBS (pH 7.2), fixed with 0.5% glutaraldehyde (PBS, pH 7.2), and washed in PBS supplemented with 1 mM MgCl_2_. Cells were stained in X-gal solution (1 mg/ml), 0.12 mM K_3_Fe [CN] _6_, 0.12 mM K_4_Fe [CN] _6_, 1 mM MgCl_2_ in PBS at pH 6.0) overnight at 37°C and observed using Nikon eclipse TS100 phase contrast microscope.

### Co-culture experiment

For, co-culture experiments, the monocytes were isolated as described earlier and plated out at 1 × 10^6^ cells/well in 24-welled plates as triplicates, treated with rapamycin for 2 h, and then washed with PBS. Furthermore, these monocytes were subjected to rWmhsp60 stimulation for 24 h. The monocytes (10^4^ cells/ml) were then co-cultured with monocyte-depleted PMBC (10^6^ cells/ml) cells in a final volume of 500 μl medium in 24-well plates and stimulated with PHA, (5 μg/ml) for 48 h. [^3^H] Thymidine from DuPont NEN (Boston, MA) was added to the cells, at a final concentration of 1 μCi/ml, 3 h prior to the termination of the experiment. The cells were harvested, and the cell lysates were put onto the filter (Glass microfibre filters; Whatman International Ltd, Maidstone, England) and were washed three times with 5% trichloracetic acid and one time with acetone on a filter. The cell pellet on the filter was dissolved in 10 ml of Biofluor (Fisher Scientific, Pittsburgh, PA), and the radioactivity was determined by liquid scintillation counting. The cell growth was expressed as stimulation index.

### Statistical analysis

Data analyses were performed using GraphPad PRISM (GraphPad Software, Inc., San Diego, CA). Mean ± SD were used for measurements of central tendency. Comparisons were made using either the Kruskal–Wallis (non-parametric ANOVA) test with Dunn’s multiple comparisons (unpaired comparisons) or the Wilcoxon signed-rank test (paired comparisons, “*” denotes p < 0.05.

## Results

### Production of recombinant Wmhsp60

**Fig 1:**
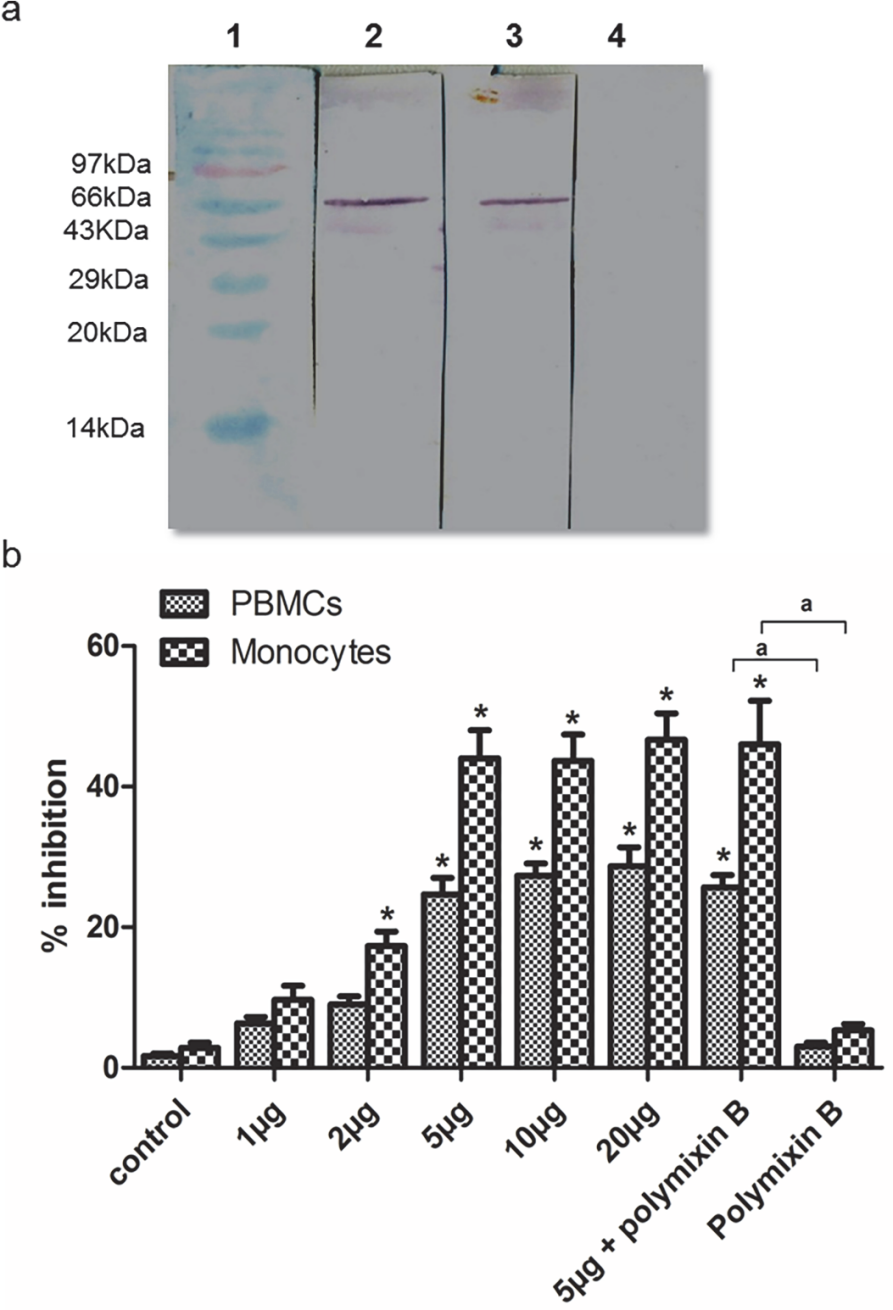
(a) Production of recombinant Wmhsp60. Western blot of recombinant and IMAC-purified Wmhsp60. Lane 1, molecular weight marker; lane 2, His-tagged rWmhsp60 probed with anti-rWmhsp60 antibody; lane 3, His-tagged rWmhsp60 probed with anti-histidine monoclonal antibody; lane 4, His-tagged rWmhsp60 probed with normal mouse serum. (b) rWmhsp60 inhibited the growth of PBMCs and monocytes in a dose-dependent manner. PBMCs and monocytes of EN were treated with different concentrations of rWmhsp60. After 48 h of incubation, cells were harvested, and proliferative effect was determined by MTT assay as described in Methods section. The optimum concentration of rWmhsp60 was found to be 5μg / 1 million cells / ml. To ensure endotoxin-free preparation of rWmhsp60, Polymyxin-B assay was performed as described in—Methods section with the optimum concentration. Values were represented as % inhibition (mean ± SD, n = 10; “*” and “^**a**^” denote significance [p<0.05]).

The *B*. *malayi Wolbachia hsp60* gene was amplified from the genomic DNA of *B*. *malayi*, cloned in pRSET-A (Life technologies), and the rWmhsp60 was purified by IMAC as reported previously [21] (Fig 1A). Sera from patients with active microfilaria and chronic pathology reacted with rWmhsp60 in Western blot, whereas sera from asymptomatic endemic and non-endemic normal individuals did not exhibit any reactivity [21].

### Cytotoxic effects of rWmhsp60

To investigate the effect of rWmhsp60 on PBMCs and monocytes, cells were cultured in 96-well plates at a concentration of 2 × 10^5^ cells/ml and treated with 1, 2, 5, 10 and 20 μg/ml of rWmhsp60 for 48 h. MTT assay revealed a dose-dependent increase in inhibition until 5 μg/ml of rWmhsp60 (Fig 1B). Further increase in the concentration did not have any significant influence in inhibition. Cytotoxicity observed with rWmhsp60 (5μg/ml) in the presence and absence of polymyxin-B sulfate indicated that the contaminating LPS did not contribute to the proliferative responses and the observed immune regulatory effect of rWmhsp60 was not due to LPS contamination.

### rWmhsp60-induced apoptosis in PBMCs of EN and CP

**Fig 2:**
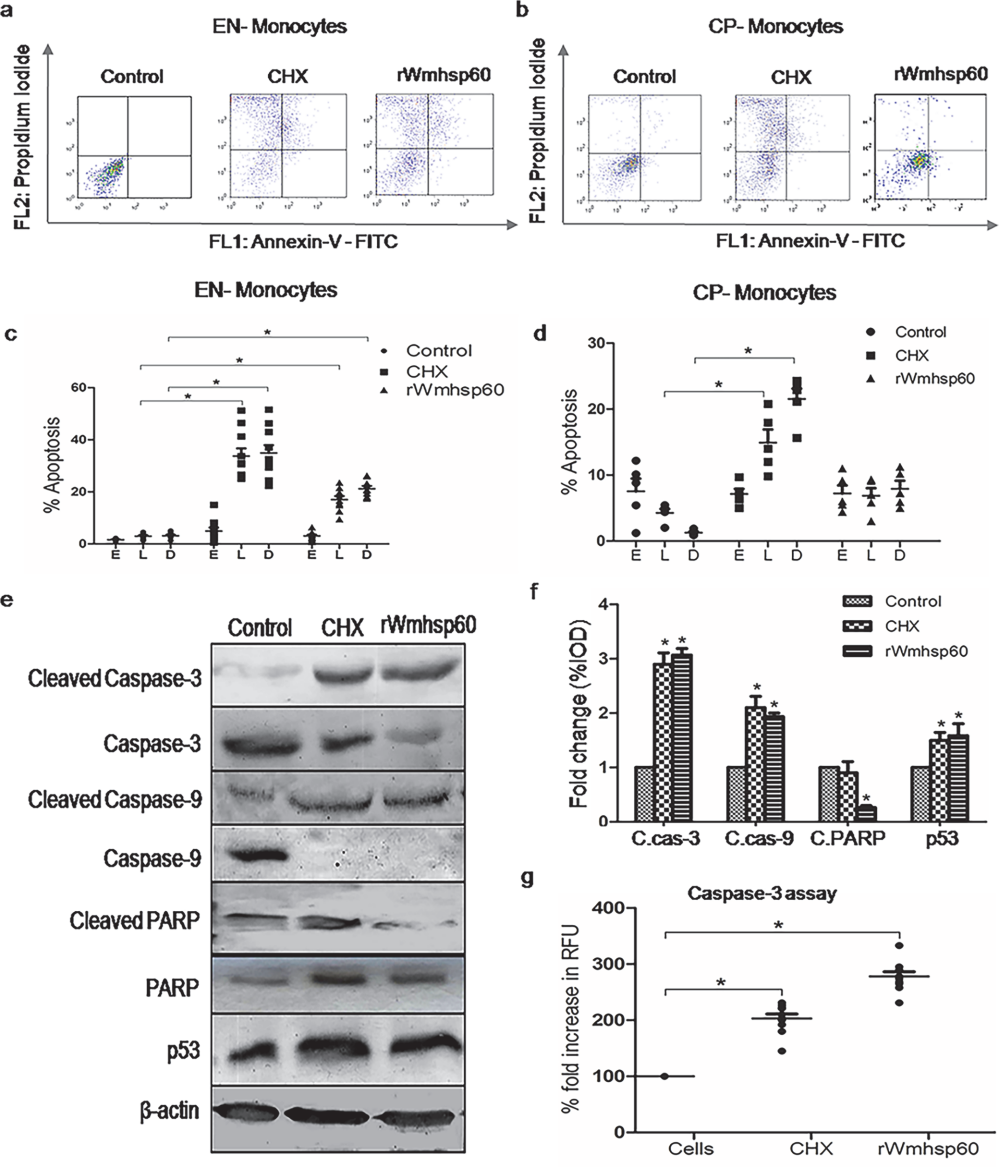
Quantitative analysis of apoptosis in PBMCs. Representative distributions of the fluorescence intensity of annexin-V-FITC and PI binding of (a) EN (n = 10) and (b) CP (n = 5) monocytes after 24-h stimulation with rWmhsp60 and CHX. Early apoptotic cells (E) were annexin-V-FITC^+^/PI^−^, late apoptotic cells (L) were annexin-V-FITC^+^/PI^+^ and dead cells (D) were annexin-V-FITC^−^/PI^+^. The scatter plot represents the percentage of early, late apoptotic and dead cells in (c) EN and (d) CP monocyte populations. Values are represented as mean ± S.D and “*” denotes p<0.05. rWmhsp60 induces apoptosis in monocytes of EN via caspase cascade activation. (e) rWmhsp60- and CHX-treated monocytes from EN were harvested, and the levels of caspase-3, caspase-9, p53 and PARP were determined by Western blot analysis as described in Methods section with the respective cleaved and total antibodies. (f) Bar graph represents the fold change of normalized IOD of the respective blots. (g) Monocytes of EN were cultured in the presence of CHX and rWmhsp60 for 24 h and determined the caspase-3 activity using the fluorogenic substrate Ac-DEVD-AMC as detailed in Methods section. The activity expressed as % fold increase in relative fluorescence units (RFU) (mean ±S.D, n = 10) and “*” represents p<0.05.

Exposure of phosphatidyl serine on the outer membrane, a key event during apoptosis, was evaluated by annexin-V-FITC staining using flow cytometric analysis. Lymphocytes and monocytes were gated separately from PBMCs based on scattering of light. In order to exclude the inner membrane staining, cells were also stained with PI. Early apoptotic cells (E) were annexin-V-FITC^+^/PI^−^, late apoptotic cells (L) were annexin-V-FITC^+^/PI^+^ and dead cells (D) were annexin-V-FITC^−^/PI^+^. Although, there was marginal increase (7%) in early apoptotic monocytes from CP patients, rWmhsp60 treatment, however, did not induce any significant change in early, late apoptotic and dead monocytes. In contrast, monocytes of EN exhibited an increase in late (18%; p = 0.0027) apoptotic and dead cells (23%; p = 0.0031) on rWmhsp60 stimulation compared with the untreated monocytes (Fig 2A, 2B, 2C and 2D). As expected, CHX treatment resulted in elevated levels of late apoptotic and dead cells in monocytes of both EN (28%; p = 0.00317 and 30%; p = 0.00293) and CP (15%, p = 0.0154 and 22%, p = 0.00197). Interestingly, lymphocytes did not show any signs of apoptosis in both EN and CP [9], thus establishing the effect of rWmhsp60 as restricted to monocytes of EN. Hence, the mechanism of rWmhsp60-induced apoptosis was assessed using monocytes of EN in further experiments.

Most apoptotic cell death process is associated with the activation of complete caspase cascade and p53 levels. Hence, to explore the caspase cascade activation events in monocytes of EN, rWmhsp60-treated monocyte cell lysates were subjected to immunoblot analysis with antibodies to cleaved and total Caspase-3, Caspase-9, PARP and p53. Prominent increase in the cleaved forms of casapase-3, caspase-9 and PARP compared with the total forms provide strong evidence that rWmhsp60 induces apoptosis via caspase-dependent mechanism. Further, western blot analysis exhibited increased expression of p53 on rWmhsp60 treatment (Fig 2E and 2F). A similar trend was also observed with CHX treatment on monocytes of EN.

As caspase-3 is a key effector molecule in apoptosis activation, we investigated its enzymatic activity using AC-DEVD-AMC caspase-3 fluorogenic substrate. rwmhsp60 treatment of monocytes from EN for 24 h markedly increased (2.3 fold; p = 0.0028) the proteolytic activity of caspase-3 as assessed using AC-DEVD-AMC fluorogenic substrate (Fig 2G). Also, CHX stimulation significantly increases the caspase-3 activity in EN (2.5 fold; p = 0.0046) as analyzed using AC-DEVD-AMC substrate. Collectively, these results augment that rWmhsp60 induces apoptosis in monocytes of EN via caspase-dependent mechanism.

### rWmhsp60 enhances ROS and mROS production in monocytes

**Fig 3:**
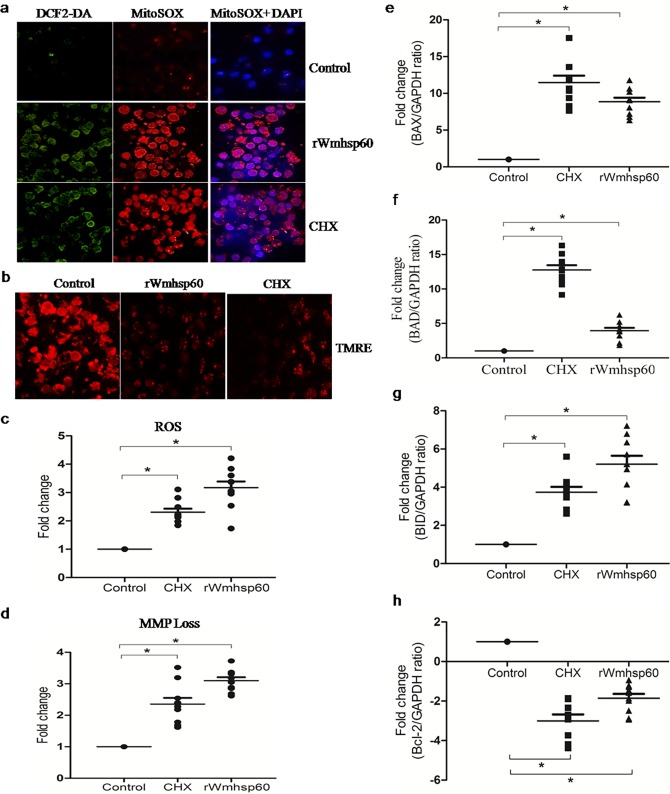
rWmhsp60 induces ROS and mROS in monocytes of EN. (a) The generation of intracellular and mitochondrial ROS in monocytes of EN with CHX and rWmhsp60 was analyzed using DCF2-DA and MitoSOX staining. Representative images showed ROS and mROS production by confocal microscopy. (b) The rWmhsp60- and CHX-induced mitochondrial membrane dissipation was observed in confocal microscope using TMRE. (c) and (d) represents quantitative fluorimetric analysis of ROS and MMP using DCF2-DA and TMRE, respectively. The bar graph represents average mean ±SD of fold change in RFU (n = 10) and * denotes p<0.05. rWmhsp60 resulted in increased expression of pro-apoptotic markers. Monocytes of EN (n = 10) were stimulated with CHX and rWmhsp60 for 24 h and harvested, and the gene expression of pro- and anti-apoptotic markers (e) *Bax*, (f) *Bad*, (g) *Bid* and (h) *Bcl-2* determined by Real Time PCR is depicted as fold change over basal level (mean ± SD; “*” represents p<0.05).

ROS signals were known to control variety of responses including apoptosis. To determine its relationship in rwmhsp60-mediated apoptosis, we assessed the spontaneous ROS and mROS generation in monocytes of EN followed by the change in ROS production after rWmhsp60 treatment using DCF2-DA and MitoSOX staining. The results revealed that the EN monocytes had spontaneous basal levels of ROS and mROS, and rWmhsp60 and CHX stimulation increased ROS and mROS production (Fig 3A) as evident from the increased number DCF2-DA and MitoSOX-stained monocytes.

As mitochondria play an important role in the intrinsic pathway of apoptosis, mitochondrial membrane potential was quantified using tetramethylrhodamine ethyl ester (TMRE). rWmhsp60 treatment resulted in increased number of EN monocytes with depolarized mitochondria compared with the control monocytes which had the intact mitochondrial integrity (Fig 3B).

The fluorimetric analysis with DCF2-DA confirmed that rWmhsp60 (3-fold; p = 0.0029) and CHX (2-fold; p = 0.0015) stimulations resulted in increased ROS levels in monocytes of EN (Fig 3C). Also, quantitative analysis with TMRE (Fig 3D) resulted in loss of mitochondrial membrane potential on rWmhsp60 stimulation (3-fold; p = 0.0085). As expected, CHX stimulation increased the depolarization of mitochondria in monocytes of EN (2-fold; p = 0.0043).

In addition, to determine whether the increased or decreased uptake of TMRE were due to the differences in the mitochondrial density or originates from the changes in the membrane integrity, the gene expression of the intrinsic pathway markers were studied. Our analysis revealed a marked increase in pro-apoptotic genes (Fig 3E, 3F and 3G) *bax* (8-fold; p = 0.0057), *bad* (2.5-fold; p = 0.0039) and *bid* (5-fold; p = 0.0028), respectively. Also, relatively decreased expression of anti-apoptotic gene (Fig 3H), *bcl-2* (3 fold; p = 0.0037), was observed on rWmhsp60 stimulation. A similar trend was observed with CHX treatment, bax 10-fold (p = 0.0017), bid 3-fold (p = 0.0034), bad 13-fold (p = 0.0019) and bcl-2 0.8-fold (p = 0.0043). In conclusion, these results suggest that increase in ROS, mROS and loss in mitochondrial membrane integrity may contribute to rWmhsp60-mediated apoptosis.

### ROS mediates rWmhsp60-induced NF-κB translocation

**Fig 4:**
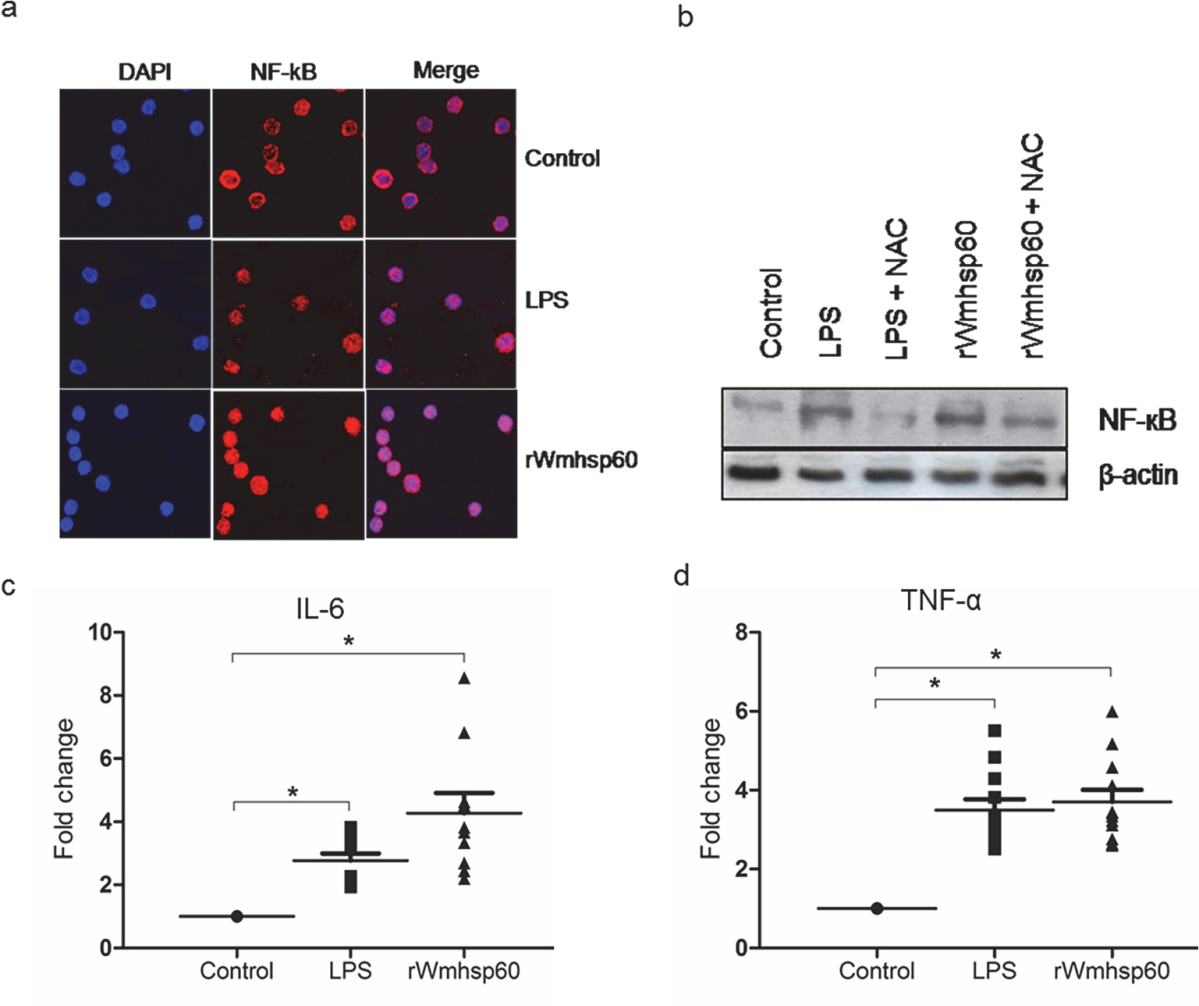
ROS production is required for rWmhsp60-induced NF-κB activation. (a) Immunofluorescence of monocytes from EN treated with rWmhsp60 and LPS indicated the localization of NF-κB p65 (red fluorescence) at ×1000 magnification. DAPI (blue) was used as a nuclear counter stain. (b) The effect of ROS on rWmhsp60-induced NF-κβ activation was analyzed in monocytes of EN (n = 10) by Western blot and β-actin. IL-6 (c) and TNF-α (d) cytokine levels in the supernatant were quantified by ELISA using specific monoclonal primary antibodies and developed using chemiluminescence and expressed as fold change (mean ± SD; “*” represents p<0.05).

To examine the effect of rWmhsp60-mediated ROS on nuclear translocation of NF-κB, the immunofluorescence staining of NF-κB-p65 was performed using confocal microscopy to provide the exact location of NF-κB in rWmhsp60 and LPS-treated and-untreated monocytes. Nuclear translocation of NF-κB was observed in EN monocytes treated with rWmhsp60 and LPS (Fig 4A). In contrast, NF-κB resides predominantly in the cytoplasm of the untreated monocytes and, thus, confirms the activation and translocation of NF-κB on rWmhsp60 stimulation.

Further to examine the effect of ROS generation on NF-κB, Western blot analysis was performed following concurrent incubation of monocytes with NAC for 1 h. Increase in the NF-κB levels were observed on rWmsp60 treatment, whereas NAC greatly inhibited NF-κB production (Fig 4B). These data suggest that ROS influence NF-κB transcriptional activation following rWmhsp60 stimulation.

Expression of TNF-α and IL-6 was regulated by transcription factor, NF-κB. To determine whether rwmhsp60 can modulate TNF-α and IL-6 production, culture supernatants of rWmhsp60-treated and untreated monocytes were assayed for TNF-α and IL-6 protein levels by ELISA. As expected, rWmhsp60 stimulation increased the production of IL-6 (4.2-fold; p = 0.005) (Fig 4C) and TNF-α (3.7-fold; p = 0.029) (Fig 4D) comparable with LPS-induced IL-6 (3 fold; p = 0.0061) and TNF-α (3.5 fold; p = 0.0089) production. Taken together, these results suggest that ROS and mROS mediate NF-κB translocation into nucleus and initiate the production of pro-inflammatory cytokines TNF-α and IL-6.

### Signaling intermediates in rWmhsp60-induced NF-κB activation: role of TLRs

**Fig 5:**
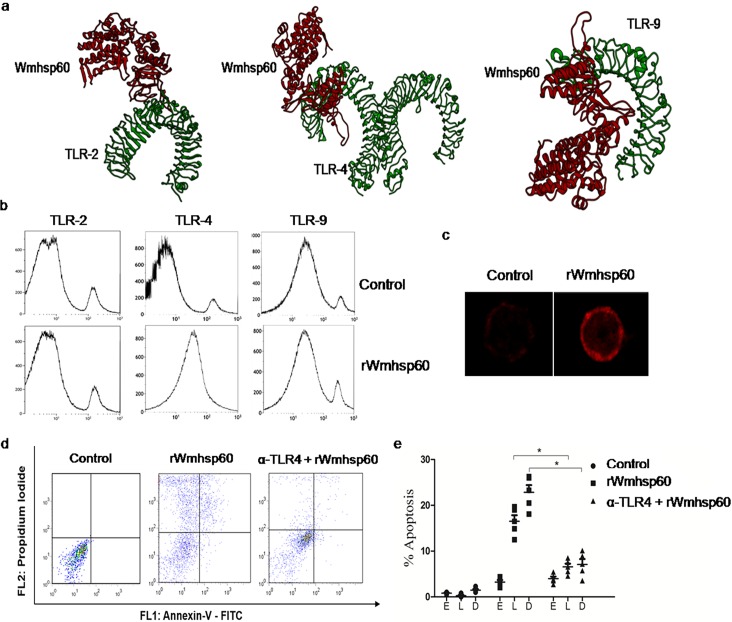
TLR4 signaling accounts for rWmhsp60-induced apoptosis. (a) Molecular docking of protein–receptor interface among TLR-2, TLR-4 and TLR-9 with rWmhsp60. (b) Surface expression of TLR-2, TLR-4 and TLR-9 in monocytes from EN with rWmhsp60 was evaluated by FCM analysis. (c) Monocytes were stimulated with rWmhsp60 and CHX and labeled with anti-TLR-4-APC antibody. Representative images showed TLR-4 surface expression by confocal microscopy (×1000). (d) Monocytes treated with rWmhsp60 and/or anti-TLR-4 blocking antibody was subjected to annexin-V-FITC/PI staining. Early apoptotic cells were annexin-V-FITC^+^/PI^−^, late apoptotic cells were annexin-V-FITC^+^/PI^+^ and dead cells were annexin-V-FITC^−^/PI^+^. (e) The statistical plots represent the percentage apoptotic and dead cells (n = 10; mean ± SD; * represents p<0.05).

To determine the mechanism by which rWmhsp60 activates the apoptotic cascade via ROS, we examined the possible role of TLRs by docking Wmhsp60 with TLR-2, TLR-4 and TLR-9 using HEX and Cluster pro-docking server. We predicted the structure of Wmhsp60 using homology-modeling approach with SWISS-PDB. TLR-2, TLR-4, and TLR-9 3D structures were obtained from RCSB. As a result, Wmhsp60 fits precisely with the binding moiety of TLR-4 with the lowest binding energy of about −440kcal (HEX) and −882.6 kcal (Cluspro) and not with TLR-2 and TLR-9 (Fig 5A). Also, non-availability of the complete structure of TLR-2 and TLR-9 was a great hindrance to make further assessment. In addition, the surface expressions of TLR-2, TLR-4 and TLR-9 on rWmhsp60 stimulation were assessed with fluorescent-tagged antibodies using flow cytometer. As shown in the Fig 5B, exposure of human monocytes for 2 h to rWmhsp60 dramatically increased the surface expression of TLR-4 and not the TLR-2 and TLR-9. Furthermore, increased TLR-4 surface expression in the rWmhsp60-treated monocytes was supported and confirmed by confocal microscopy studies (Fig 5C).

To confirm that rWmhsp60 was, indeed, signaling through TLR-4, we pre-treated monocytes with anti-TLR-4 monoclonal antibodies, 2 h before stimulation with rWmhsp60. Anti-TLR-4 pretreatment significantly reduced the late apoptotic (6%; p = 0.0075) and dead cells (6.5%; p = 0.0084) compared with rWmhsp60 treatment that exhibited increased late (18%; p = 0.0058) apoptotic and dead cells (23%; p = 0.0029) (Fig 5D and 5E). Collectively, these experiments synergistically augment that rWmhsp60-induced monocyte apoptosis is triggered via TLR-4 signaling pathway.

### rWmhsp60-induced autophagy and senescence in monocytes

**Fig 6:**
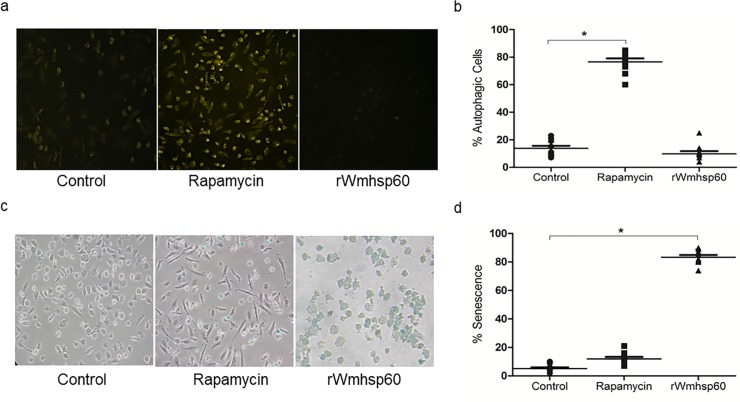
rWmhsp60-induced autophagy and senescence. (a) Visualization of autophagy activation with MDCin monocytes of EN. Cells treated with rWmhsp60 and rapamycin were incubated for 24 h. Cells were stained with MDC as described in Methods section and visualized under fluorescent microscope at 335 nm. (b) The percentage of MDC-stained cells was represented as % autophagic cells (n = 10; mean ±SD; “*” represents p<0.05). (c) rWmhsp60 stimulation potentiates senescence in monocytes of EN. Monocytes were treated as above and incubated overnight in the presence of X-Gal (1 mg/ml) at 37°C as described in the Methods section. SA-β-Gal activity was detected by a blue cell staining, visualized under Carl Zeiss inverted microscope, and analyzed using Axiovision software. (d). SA-b-Gal–positive cells were quantified by counting 10^2^ cells on three separate fields for each condition. The bar graph represents average mean ± SD of fold changes (n = 10) and “*” denotes p<0.05.

In attempt to characterize the mechanism by which rWmhsp60 induces cell death other than apoptosis, we have examined for autophagy and senescence in rWmhsp60-treated monocytes. To detect autophagy, rWmhsp60 stimulated monocytes were subjected to MDC staining. Rapamycin-treated monocytes that served as a positive control exhibited high intensities (75%; p = 0.0015) of MDC staining (Fig 6A and 6B), whereas rWmhsp60 stimulated monocytes that failed to uptake MDC and remained as similar to control monocytes, suggesting that rWmhsp60 does not induce autophagy in monocytes. Senescence was characterized as SA-β-Gal–positive cells. Increase in SA-β-Gal–positive cells (85%; p = 0.0019) were detected in response to rWmhsp60 treatment (Fig 6C and 6D) versus 2% in control cells. As expected, rapamycin does not show any significant increase in SA-β-Gal–positive cells (8%). This confirms that rWmhsp60 induces monocyte senescence in addition to apoptosis.

### Rapamycin-triggered autophagy mediates protection

**Fig 7:**
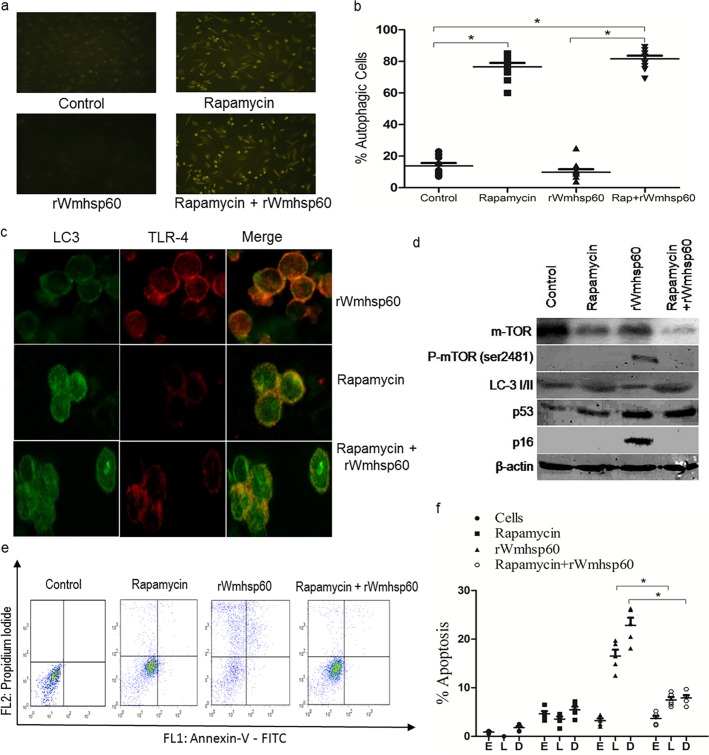
Rapamycin protects monocytes of EN from rWmhsp60-induced apoptosis. (a) Monocytes were treated for 24 h with rWmhsp60 following 2 h incubation with or without rapamycin. Further, cells were stained with MDC as described in Methods section and visualized under fluorescent microscope at 335 nm. (b) The percentage of cells with MDC stained was represented as % autophagic cells. Rapamycin targets TLR4 to autophagosome. (c) Intracellular vesicle co-localization of TLR4 into autophagosome (LC3 positive: 3 h) following rapamycin and/or with rWmhsp60 treatment was assessed as described in the Methods section and visualized using confocal microscopy (×1000). (d) Immunoblot analysis was performed to assess the expression of LC3, mTOR, p16 and p53 in rapamycin pre-treated monocytes of EN following rWmhsp60 stimulation. β-actin was used as a loading control. (e) Representative distributions of the fluorescence intensity of annexin-V-FITC and PI binding of EN monocytes following rWmhsp60 and or rapamycin treatment. (f) The statistical plots represent the percentage apoptotic and dead cells (n = 10; mean ± SD; “*” represents p<0.05).

In order to reduce the p53-mediated apoptosis and senescence-induced inflammation in rWmhsp60-stimulated monocytes, we hypothesized to induce p53-mediated autophagy using rapamycin prior to rWmhsp60 treatment. Also, rapamycin is known to sequester and degrade the pro-inflammatory TLR signaling complex in autophagy-dependent manner. Rapamycin-treated monocytes served as a positive control that exhibited high intensities (75%; p = 0.0075) of MDC staining, whereas rWmhsp60-stimulated monocytes failed to uptake MDC and remained as similar to control monocytes (Fig 7A and 7B). In addition, rWmhsp60 stimulation of monocytes post-rapamycin treatment (82%) does not influence any change in MDC staining compared with rapamycin-treated monocytes. This clearly suggests that rapamycin-induced autophagosome formation in monocytes was not altered on rWmhsp60 stimulation.

In addition, we investigate the localization of TLR-4 on rapamycin and rWmhsp60 treatment using Confocal microscopy. Indeed, rWmhsp60 stimulation does not induce vesicular localization of TLR-4 and LC3, whereas rWmhsp60 stimulation following rapamycin pretreatment exhibited co-localization of LC3 with TLR-4. Also, it is evident from the Fig 7C that rapamycin pretreatment sequesters TLR-4 into endosomal vesicle and degrades the TLR signaling cascade. As a result, rWmhsp60 fails to activate TLR signaling cascade following rapamycin treatment. Similarly, Western blot analysis revealed that the expression of mammalian target of rapamycin (m-TOR) by rWmhsp60 was abrogated by rapamycin pretreatment (Fig 7D). Furthermore, expression of LC-3 an autophagy marker following rapamycin treatment was not altered by rWmhsp60. Thus, this study provides the evidence of involvement of autophagy in rapamycin-induced hyporesponsiveness to rWmhsp60 and is mediated by degradation of TLR-4.

Furthermore, to analyze, rapamycin pretreatment protects rWmhsp60-induced apoptosis in EN monocytes, annexin-V/PI analysis was performed (Fig 7E). As expected, EN monocytes exhibited increased late (18%; p = 0.0027) apoptotic and dead cells (23%; p = 0.0031) on rWmhsp60 stimulation. In contrast, rapamycin treatment prior to rWmhsp60 stimulation significantly decreased the late (6.5%; p = 0.0017) apoptotic and dead (7.5%; p = 0.0025) cells. Thus, rapamycin significantly reduced the rWmhsp60-induced apoptosis by 27% (annexin V, annexin V + PI and PI-positive cells; Fig 7F).

**Fig 8:**
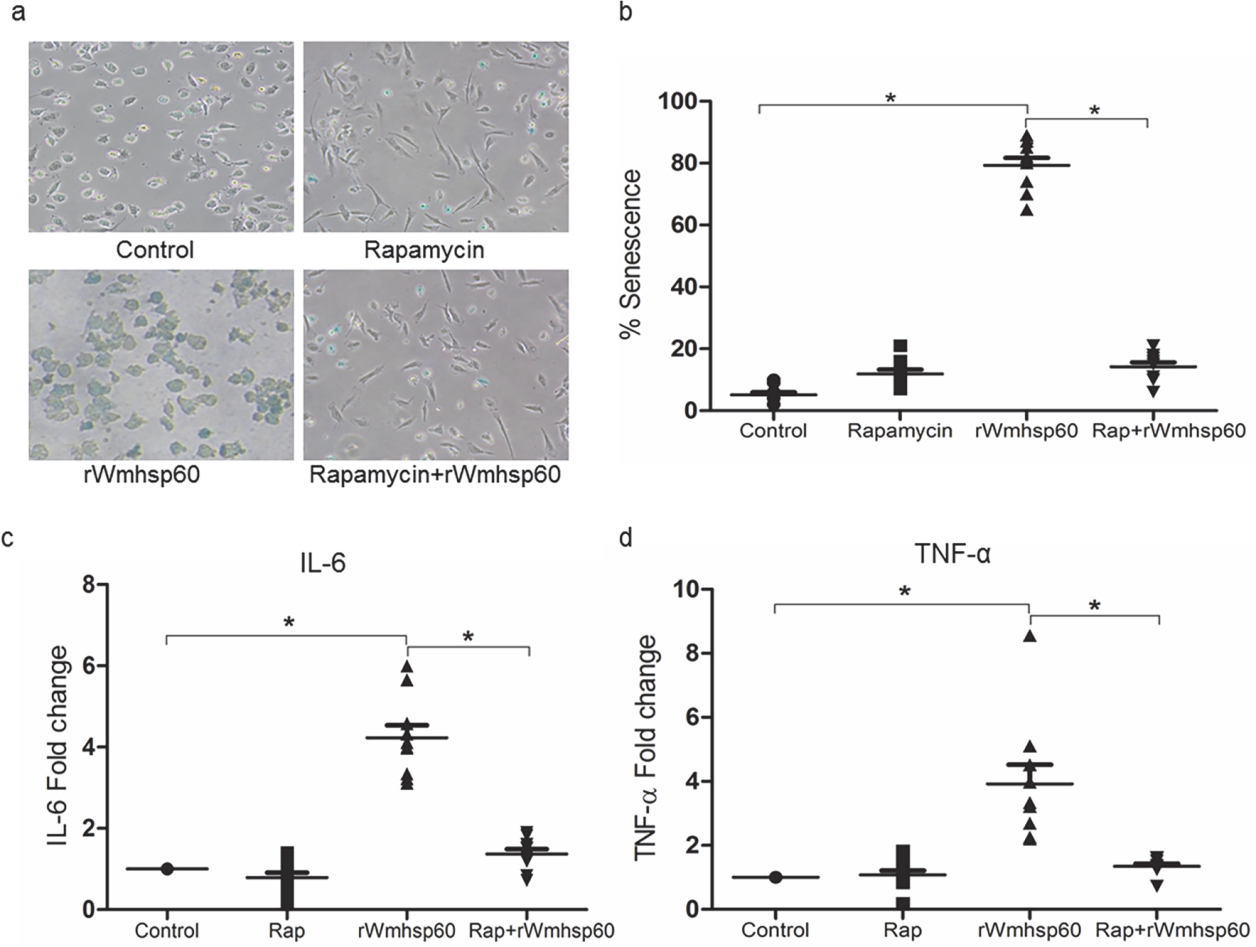
Rapamycin protects monocytes of EN from rWmhsp60 induced senescence and inflammation. (a) Monocytes treated with rWmhsp60 and/or rapamycin were subjected to SA-β-Gal activity staining as stated earlier and acquired in Carl Zeiss inverted microscope and analyzed using Axiovision software (Carl Zeiss, Jena). (b) SA-β-Gal–positive cells were quantified by counting 10^2^ cells on three separate fields for each condition. IL-6 (c) and TNF-α (d) cytokine levels in the supernatant were quantified by ELISA using specific monoclonal primary antibodies and developed by chemiluminescence and expressed as fold (n = 10; mean ± SD; “*” represents p<0.05).

Similarly, increase in SA-β-Gal–positive cells (78%; p = 0.0026) were detected in response to rWmhsp60, whereas rapamycin pretreatment decreased the SA-β-Gal–positive cells to 10% (p = 0.086; Fig 8A and 8B). Further, to determine whether rapamycin can modulate rWmhsp60-induced pro-inflammatory TNF-α and IL-6 production, culture supernatants of EN monocytes treated with rapamycin and/or rWmhsp60 were assayed for TNF-α and IL-6 protein levels by ELISA. As expected, rWmhsp60 stimulation resulted in perceptible increase in the production of IL-6 (4.2 fold; p = 0.005) and TNF-α (3.7 fold; p = 0.029) (Fig 8C and 8D). In contrast, rWmhsp60-stimulated monocytes post-rapamycin treatment significantly decreased TNF-α and IL-6 protein levels comparable with the control. Collectively, these results demonstrate that autophagy protects monocytes from rWmhsp60-induced apoptosis and senescence by the induction of autophagy. Finally, MDC and β-Gal staining revealed that rapamycin also induces differentiation of monocytes.

### Activation of lymphocytes by rapamycin-pretreated monocytes

**Fig 9:**
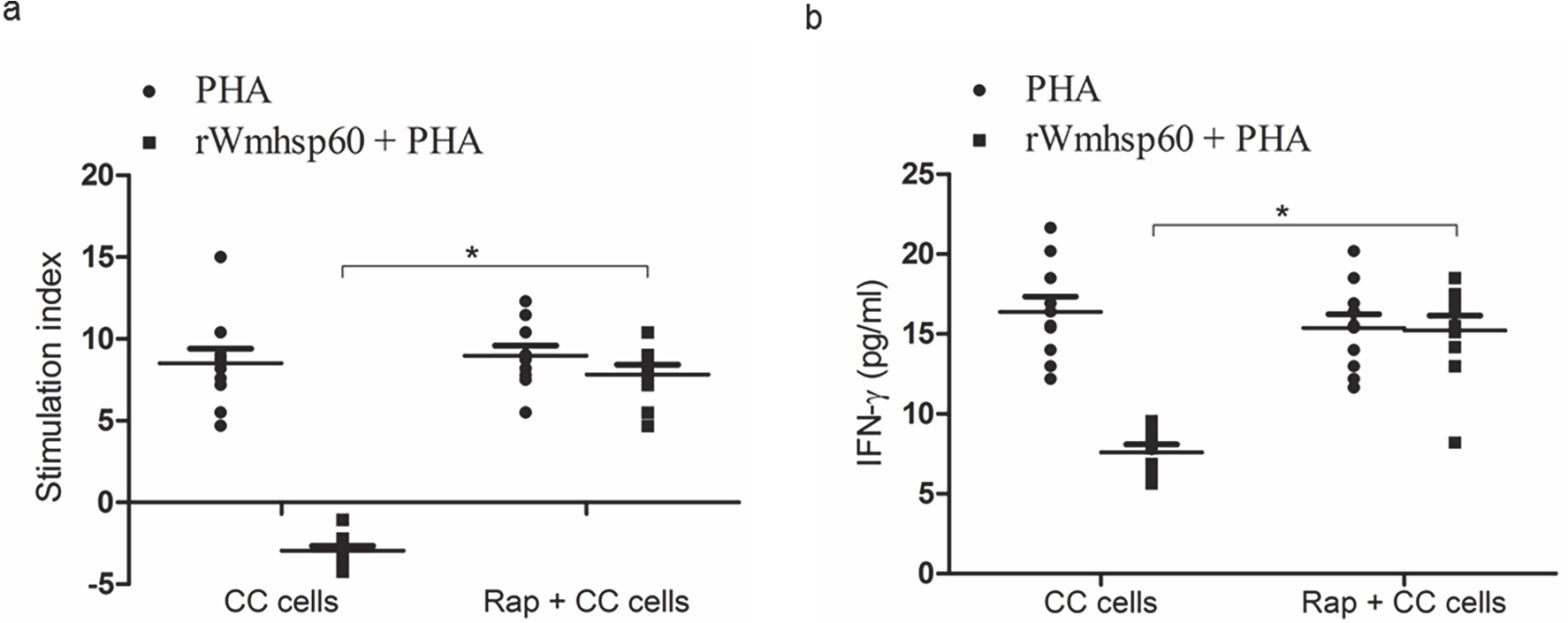
Activation of lymphocytes by rapamycin-treated monocytes from EN. Monocytes of EN were purified from PBMCs by percol gradient method and treated with rapamycin for 2 h. Rapamycin-treated monocytes were then co-cultured with monocyte depleted PBMCs (CC cells). (a) After 24 h, assessment of lymphocyte proliferation using [^3^H] thymidine was carried out following PHA treatment with or without rWmhsp60. Lymphocyte proliferation was expressed as stimulation index. (b) The concentration of IFN-γ in the cell culture medium was determined by enzyme-linked immunosorbent assay as described in Methods section (n = 10; mean ± S.D and * denotes p<0.05).

Finally, we investigated whether rapamycin treatment on monocytes can activate lymphocytic cells. As expected, co-culturing of monocytes with monocyte-depleted PBMCs and rapamycin-treated monocytes with monocyte-depleted PBMCs resulted in the similar levels of proliferation to PHA stimulation. However, co-culturing of rWmhsp60-treated monocytes abrogated the PHA-induced proliferation of monocyte-depleted PBMCs. Nevertheless, rapamycin pretreatment to the monocytes reversed this effect and showed a 9-fold (p = 0.019) increase in the proliferation following PHA stimulation (Fig 9A). Furthermore, increase in proliferation was substantiated with increase in IFN-γ levels (Fig 9B) in the culture supernatants of the above-mentioned stimulated cultures. These results suggest that rWmhsp60 downregulates T-cell activation by monocytes and rapamycin pretreatment abrogated this effect.

## Discussion

*Wolbachia* in filariae is gaining importance as it is implicated with inflammatory responses and pathogenesis of filarial infections [30]. More precisely, adverse inflammatory reactions and diminished APC function, attributed during disease conditions, are also observed with Wolbachial antigens [11–13]. This could be one among many existing explanation for T-cell hyporesponsiveness, a hallmark status in filarial pathogenesis. However, till date, there is dearth of proper evidence in its support to unravel the probable potentials of Wolbachial antigens in filarial pathogenesis. Consequently, sensing the gravity, we earlier demonstrated that Wolbachial antigens (hsp60 and WSP) induce T-cell suppression and monocyte apoptosis in normal healthy population [9,22]. Similar T-cell anergy [4] and impaired monocyte functions [6–8] were reported earlier in filarial patients; however, the molecular mechanisms underlying these functions remain elusive. Hence, the present study was particularly designed to account for this lacuna by investigating Wolbachial hsp60-modulated apoptosis in endemic normals and CPs.

Flow cytometric analysis with annexin-V and PI has revealed that monocytes of EN were more susceptible to rWmhsp60-induced apoptosis, whereas lymphocytes of EN and CP and monocytes of CP exerted resistance to rWmhsp60-induced apoptosis, thus establishing its specificity to monocytes of EN. Possible reason for this differential susceptibility between monocytes of EN and CP could be due to the binding ability of hsp60 to TLR [31] and suppressed expression of TLRs in CP [32]. Resistance offered by lymphocytes of EN and CP may be due to lesser expression of TLRs in lymphocytes in comparison with monocytes [33,34]. Similar monocyte apoptosis mediated by filarial antigens was previously reported [35,9]. This prompted us to elucidate the mechanism of rWmhsp60-mediated apoptosis in monocytes of EN.

Recent studies with Wolbachial surface antigen by Brattig and studies with other hsps limited our search for hsp’s surface receptors to TLR-2, -4 and -9 [23]. In this report, we showed that rWmhsp60 can interact with TLR-4, by the upregulation of TLR-4 but not TLR-2 and TLR-9 surface expression, confirmed by flow cytometric analysis and confocal microscopy. In addition, blocking experiments with anti-TLR4 antibodies inhibited apoptosis, proving a sharp note that rWmhsp60 induces apoptosis via TLR-4. Furthermore, docking studies with TLR-4 also provided evidence that Wmhsp60 can directly bind to the TLR-4. These results are in broad agreement with the demonstration of TLR-4 as a receptor for human hsp60 [31]. Apart from identifying receptor for Wmhsp60, the present study to acquire insights into molecular events in activation pathway of monocyte will be of crucial interest since the nature of the ligand determines the phenotype of activation. There are enormous previous evidences suggesting that ROS can act as a secondary messenger [36] and control various signaling molecules downstream of TLR [37]. Increased ROS and mROS levels and altered mitochondrial bioenergetics were observed in apoptotic monocytes as a result of Wmhsp60–TLR-4 interaction. Apoptosis mediated by mitochondrial membrane potential loss was regulated and controlled by *Bcl-2* family of pro-apoptotic *Bax*, *Bid* and *Bad* and anti-apoptotic *Bcl-2* markers. An elevation in the levels of pro-apoptotic markers relative to concomitant *Bcl-2* expression in rWmhsp60-treated monocytes confirms that apoptosis is through mitochondria-mediated pathway. Similarly, activation of caspases, the major downstream events following mitochondrial membrane potential loss, is responsible for many of the molecular changes in the cell undergoing apoptosis [38]. Activation of caspase cascade and PARP degradation by rWmhsp60 provided the strong evidence that the cell death is initiated via mitochondrial disruption pathway and caspase cascade activation. There are numerous reports supporting this mechanism. Furthermore, there are few evidences on ROS contribution to translocation and transcriptional activation of NF-κB [39]. In our study, rWmhsp60 stimulation led to the translocation of NF-κB into the nucleus of monocytes. In addition, we found that rWmhsp60-induced consistent expression pattern of NF-κB was abrogated on NAC treatment, which suggests the role of ROS in NF-κB production and translocation. Furthermore, NF-κB translocation resulted in transcriptional activation of pro-inflammatory cytokines TNF-α and IL-6. Excessive release of TNF-α may further lead to apoptosis via TNF-R [40]. Hence, both the death receptor and the mitochondrial pathways are likely to be involved in rWmhsp60-induced apoptosis. Altogether, these results suggest that rWmhsp60 interacts with TLR-4 and results in apoptosis mediated by ROS generation and subsequent TNF-α release. Thus, Wmhsp60-exposed monocytes undergo immune dysfunction at an early time point, reducing the window of time during which they might interact with T-cells.

p53, besides a key apoptosis regulator, is known to promote and impede autophagy and senescence by the ability to control metabolic stress through regulation of ROS and mTOR activity [41]. Elevated p53 expression confirms apoptosis and suggests that rWmhsp60 may also regulate other death mechanisms, apart from apoptosis. rWmhsp60 promotes mTOR phosphorylation, suggesting the absence of autophagy. X-gal staining and p16 expression synergistically augment that rWmhsp60 induces senescence in monocytes. This study, for the first time, reported that rWmhsp60, antigen involved in filarial pathogenesis, induces senescence in monocytes along with apoptosis. These senescent monocytes were the additional source of inflammatory factors other than apoptotic bodies that may ameliorate the degree of chronicity [41–43].

Constitutive stress by rWmhsp60 leads to persistent p53 activity that induces apoptosis and senescence, whereas pretreatment with rapamycin may tip the balance toward autophagy, favoring anti-inflammatory effects. Rapamycin is an inhibitor of m-TOR [44], downstream target of PI3K, and an important factor in TLR signaling [45]. Also, rapamycin was shown to block TLR-2—and TLR-4–mediated TNF-α and IL-6 production in neutrophils [46]. These studies, therefore, suggest that autophagy may act to limit TLR-mediated inflammatory conditions. Rapamycin pre-treatment sequesters TLR-4 into late endosomes which prevented TLR-4–rWmhsp60 interaction, thereby inhibiting TLR-4–mediated apoptosis and senescence. Furthermore, the lymphocyte activation study reveals that monocytes pretreated with rapamycin were unresponsive to rWmhsp60 and induces the proliferation of lymphocytes along with increase in IFN-γ levels. This study demonstrated that rapamycin pretreatment of monocytes addresses the key events during filarial pathogenesis and help monocytes and lymphocytes to uphold its function. This further corroborates with the earlier findings by others to limit excessive inflammation triggered by TLRs during sepsis and other inflammatory disorders [47].

Collectively, rWmhsp60 acts via TLR-4–dependent pathway to disrupt the maturation process of monocytes by inducing apoptosis and senescence. The resulting monocytes have functional defects that may have an impact on their ability to stimulate specific T-cell responses. This suggests that parasite may take advantage of this process to evade the immune response and establish its niche. As a counteractive measure, we propose that rapamycin pretreatment could help monocytes to retain its function even after rWmhsp60 exposure. The proactive interference of rapamycin instigates autophagy and subverts TLR-4 molecular signaling crosstalk with rWmhsp60. This strategy undermines inflammation-mediated via apoptosis and senescence and contributes to novel therapeutic applications. As rapamycin is specific to TLR-mediated mechanism and not to rWmhsp60, administration of rapamycin in combination with other anti-filarial drugs might help to control the adverse TLR-mediated inflammatory reactions following microfilaricidal treatment.
